# In Vitro Model Systems of Coxsackievirus B3-Induced Myocarditis: Comparison of Commonly Used Cell Lines and Characterization of CVB3-Infected iCell^®^ Cardiomyocytes

**DOI:** 10.3390/v13091835

**Published:** 2021-09-14

**Authors:** Lisa Kraft, Martina Sauter, Guiscard Seebohm, Karin Klingel

**Affiliations:** 1 Cardiopathology, Institute for Pathology and Neuropathology, University Hospital Tübingen, Liebermeisterstraße 8, D-72076 Tübingen, Germany; lisa.kraft@student.uni-tuebingen.de (L.K.); martina.sauter@med.uni-tuebingen.de (M.S.); 2 Department of Cardiovascular Medicine, Institute for Genetics of Heart Diseases (IfGH), University Hospital Münster, Robert-Koch-Str. 45, D-48149 Münster, Germany; guiscard.seebohm@ukmuenster.de

**Keywords:** myocarditis, coxsackievirus B3, iCell^®^, Cardiomyocytes, necroptosis, RIPK1, RIPK3

## Abstract

Coxsackievirus B3 (CVB3) belongs to the enteroviruses, which are a well-known cause of acute and chronic myocarditis, primarily infecting cardiac myocytes. As primary human cardiomyocytes are difficult to obtain, viral myocarditis is quite frequently studied in vitro in different non-cardiac and cardiac-like cell lines. Recently, cardiomyocytes that have been differentiated from human-induced pluripotent stem cells have been described as a new model system to study CVB3 infection. Here, we compared iCell^®^ Cardiomyocytes with other cell lines that are commonly used to study CVB3 infection regarding their susceptibility and patterns of infection and the mode of cell death. iCell^®^ Cardiomyocytes, HeLa cells, HL-1 cells and H9c2 cells were infected with CVB3 (Nancy strain). The viral load, CVB3 RNA genome localization, VP1 expression (including the intracellular localization), cellular morphology and the expression of cell death markers were compared. The various cell lines clearly differed in their permissiveness to CVB3 infection, patterns of infection, viral load, and mode of cell death. When studying the mode of cell death of CVB3-infected iCell^®^ Cardiomyocytes in more detail, especially regarding the necroptosis key players RIPK1 and RIPK3, we found that RIPK1 is cleaved during CVB3 infection. iCell^®^ Cardiomyocytes represent well the natural host of CVB3 in the heart and are thus the most appropriate model system to study molecular mechanisms of CVB3-induced myocarditis in vitro. Doubts are raised about the suitability of commonly used cell lines such as HeLa cells, HL-1 cells and H9c2 cells to evaluate molecular pathways and processes occurring in vivo in enteroviral myocarditis.

## 1. Introduction

Coxsackievirus B3 (CVB3) is a non-enveloped, positive-sense single-stranded RNA virus belonging to the enteroviruses. Enteroviruses are among the most common triggers causing myocarditis. Myocarditis is defined as an inflammatory disease of the myocardium which can progress to dilated cardiomyopathy (DCM), and ultimately to heart failure [1]. Still, no curative therapy for the treatment of viral myocarditis has been approved [1,2]. Thus, especially for the development of new therapeutic approaches, it will be critical to elucidate further the molecular mechanism of viral myocarditis in vivo and in vitro.

Optimally, the molecular mechanisms of viral myocarditis are studied in vitro in human cardiomyocytes. However, as primary human heart tissue is difficult to obtain, different cell lines are used to study CVB3-induced myocarditis. Among the most frequently used cell lines are non-cardiac cells like HeLa cells, but also cardiac-like cells like HL-1 cells or H9c2 cells [3,4,5]. HL-1 cells are cardiac muscle cells that have been derived from the AT-1 mouse atrial cardiomyocyte tumor lineage. They are supposed to show characteristics typical of embryonic atrial cardiac muscle cells but are not representative for adult human cardiomyocytes [4]. H9c2 cells have been derived from embryonic BDIX rat heart tissue and are supposed to exhibit many of the properties of skeletal muscle [6]. Although it is common knowledge that these cell lines represent human cardiomyocytes only distantly, they are used quite frequently to study cellular processes in CVB3-induced myocarditis.

Contradictory results have been published concerning the type of cell death that is caused by CVB3. The results differ in dependence on the model system being used to study CVB3 infection. Bozym et al. found that polarized intestinal epithelial cells (Caco-2 cells) perish by necrosis upon CVB3 infection [7]. In contrast, others describe cardiomyocyte apoptosis in the heart of CVB3-infected mice and in CVB3-infected HeLa and H9c2 cells [3,5,8,9,10]. Recently, the role of programmed necrosis (necroptosis) was discussed in a mouse model of viral myocarditis [11]. Necroptosis was first recognized as a caspase-independent form of cell death that can be triggered by treatment with TNF-α only in the presence of a pan-caspase inhibitor [12]. Similar to apoptosis, necroptosis is a regulated form of cell death, but leads to the typical morphological signs of necrosis such as increases in cell volume, lack of DNA fragmentation and loss of cell membrane integrity [13]. The key players for executing necroptosis are the receptor-interacting serine/threonine-kinase-1 (RIPK1) and receptor-interacting serine/threonine-kinase-3 (RIPK3). Upon activation, these kinases form a complex called the necrosome which activates the pro-necroptotic protein mixed lineage kinase domain like pseudokinase (MLKL) via phosphorylation. The phosphorylated form of MLKL was shown to be responsible for the disruption of the cell membrane’s integrity, thereby causing cell death [14,15]. Another form of programmed cell death, which is also discussed in the context of myocarditis, is pyroptosis. This highly inflammatory form of cell death is characterized by activation of caspase-1 and formation of the inflammasome [16,17].

The development of human-induced pluripotent stem cells (hiPSCs) provides unprecedented possibilities. Human cells which are difficult to obtain can now be produced at larger scales [18]. Human cardiomyocytes that have been differentiated from hiPSCs are available from different suppliers. Sharma et al. demonstrated that cardiomyocytes that have been differentiated from hiPSCs express the coxsackievirus and adenovirus receptor (CAR), which is an important prerequisite for CVB3 entry. In addition, they demonstrated that these cardiomyocytes are susceptible to infection with a luciferase-expressing CVB3 strain (CVB3-Luc), thus establishing this cardiomyocyte/CVB3-Luc system as a platform to quantitatively assess the efficacy of antiviral compounds in reducing coxsackievirus proliferation [19].

As these findings suggest that cardiomyocytes being differentiated from hiPSCs represent a suitable model system to investigate molecular mechanisms in CVB3-induced myocarditis, we aimed to compare those cells with different cell lines that are commonly used to study CVB3-induced myocarditis. The viral load, CVB3 RNA localization, VP1 expression and localization, cellular morphology in the course of infection, and the expression of several cell death markers were compared, revealing relevant differences between the various cell lines. Special emphasis was put on the mode of cell death of CVB3-infected cardiomyocytes that have been differentiated from hiPSCs (iCell^®^ Cardiomyocytes).

## 2. Materials and Methods

### 2.1. Virus

CVB3 (Nancy strain) was grown and propagated in Vero cells. Preparation of virus stocks and plaque assays were performed as described previously [20].

### 2.2. Cell Culture

HeLa cells (CCL-2) and Vero cells (CCL-81) were obtained from ATCC (Manassas, VA, USA) and cultured in Dulbecco’s modified Eagle’s medium containing 10% fetal bovine serum and 1% penicillin/streptomycin (Thermo Fisher Scientific, Waltham, MA, USA) at 37 °C and 5% CO_2_. HL-1 cells (a generous gift from Dr. William Claycomb (Louisiana State University Medical Center, New Orleans)) were cultured in full Claycomb medium (Sigma Aldrich, St. Louis, MO, USA) containing 10% fetal bovine serum, 1% penicillin/streptomycin (Thermo Fisher Scientific, Waltham, MA, USA), 0.1 mM norepinephrine (Sigma Aldrich, St. Louis, MO, USA), and 2 mM L-glutamine at 37 °C and 5% CO_2_ in gelatin/fibronectin (Sigma Aldrich, St. Louis, MO, USA) coated flasks. H9c2 cells were obtained from ECACC (via Sigma-Aldrich, Taufkirchen, Germany) and cultured in Dulbecco’s modified Eagle’s medium containing 10% fetal bovine serum and 1% penicillin/streptomycin at 37 °C and 5% CO_2_. iCell^®^ Cardiomyocytes (Donor ID number 01434, female, Caucasian, < 18 years; FUJIFILM Cellular Dynamics Inc., Madison, WI, USA) were cultured according to the manufacturer’s instructions.

For all infection experiments, cells were CVB3-infected with a MOI of 10 by adding the virus to the medium without further washes. The course of cellular morphology in infection was assessed by brightfield microscopy using an Axiovert 135 microscope (Carl Zeiss AG, Oberkochen, Germany). For inhibition experiments, cells were pre-treated for 1 h with GSK’872 (Merck Millipore, Burlington, MA, USA), necrostatin-1 (Santa Cruz Biotechnology, Dallas, TX, USA), E64d (Santa Cruz Biotechnology, Dallas, TX, USA), pepstatin A (Enzo Life Sciences, Farmingdale, NY, USA) or Z-VAD(OMe)-FMK (Cayman Chemical, Ann Arbor, MI, USA) (final concentration 100 µM, only GSK’872 was added to a final concentration of 3 µM and pepstatin A to a final concentration of 10 µM), before infection with CVB3. Control experiments for activation of caspase-3 were conducted using staurosporine (Abcam, Cambridge, UK) at a final concentration of 1 µM.

### 2.3. RNA Isolation and Quantitative RT-PCR (qRT-PCR)

RNA was isolated from CVB3-infected and uninfected cells using the RNeasy Mini Kit (Qiagen, Hilden, Germany) according to the manufacturer’s protocol. Fifty nanograms of RNA were used to perform one-step quantitative real-time reverse transcription—PCR (TaqMan RNA-to-CT 1-Step Kit, Applied Biosystems, Foster City, CA, USA) at the appropriate annealing temperature for 40 cycles on a 7300 Real Time PCR System (Applied Biosystems, Foster City, CA, USA). Data analysis was performed as absolute quantification. The number of CVB3 genomes was determined in relation to an external virus standard. Specific primers and probes were purchased from MWG Biotech (Ebersberg, Germany). 

Primers and probe were:

PanEntero: fwd: 5′ TCCTCCGGCCCCTGA 3′;

PanEntero rev: 5′ RATTGTCACCATAAGCAGCCA 3′;

PanEntero probe: 5′ FAM-CGGAACCGACTACTTTGGGTGWCCGT-TAM 3′

At least two technical replicates were assessed.

### 2.4. RNA/RNA In Situ Hybridization (ISH)

Cells were grown on microscopic slides or cover slips and CVB3 RNA was detected by in situ hybridization using RNAscope probes specific for CVB3 (Advanced Cell Diagnostics, Newark, CA, USA), followed by the RNAscope 2.5 HD Detection Kit—RED (Advanced Cell Diagnostics, Newark, CA, USA) according to the manufacturer’s protocol.

### 2.5. Western Blot

For Western blotting, CVB3-infected and uninfected cells were washed twice with PBS and lysed with ice-cold RIPA buffer (50 mM Tris-HCl (pH 7.4), 150 mM NaCl, 1% Triton X-100, 0.5% sodium deoxycholate, 0.1% SDS), supplemented with cOmplete Mini Protease Inhibitor Cocktail, (Hoffmann-La Roche, Basel, Switzerland) for 20 min on ice. Lysates were cleared by centrifugation for 15 min at 12,000 rpm and 4 °C. Heart tissue samples from patients with myocarditis were lysed with ice-cold RIPA buffer (50 mM Tris-HCl (pH 7.4), 150 mM NaCl, 1% Triton X-100, 0.5% sodium deoxycholate, 0.1% SDS) supplemented with cOmplete Mini Protease Inhibitor Cocktail, (Hoffmann-La Roche, Basel, Switzerland) for 30 min on ice, followed by sonification (twice for 15 s). Lysates were cleared by centrifugation for 15 min at 12,000 rpm and 4 °C. The extracted proteins were separated using SDS-PAGE (10% polyacrylamide gels) and subsequently blotted onto PVDF membranes. Membranes were blocked for 15 min with Antibody Diluent (Zytomed Systems, Bargteheide, Germany) and incubated with the respective primary antibody overnight at 4 °C (anti-RIP3 (Abcam, Cambridge, GB), anti-RIP, anti-Cleaved Caspase-3, anti-Cleaved Caspase-1, anti-GFP (Cell Signaling Technology, Danvers, MA, USA), anti-GAPDH (Santa Cruz Biotechnology, Dallas, TX, USA), anti-CVB3 VP1 (Mediagnost, Reutlingen, Germany)). All primary antibodies were diluted in Antibody Diluent at a 1:1000 dilution except anti-CVB3 VP1, which was diluted 1:10,000. After incubation with secondary peroxidase-conjugated goat anti-rabbit and goat anti-mouse IgG antibodies (Dianova, Hamburg, Germany; diluted 1:5000 in Antibody Diluent), chemiluminescent protein detection was performed with SuperSignal West Pico PLUS Chemiluminescent Substrate (Thermo Fisher Scientific, Waltham, MA, USA). Densitometric analysis of protein bands was performed with the ImageJ image analysis software (National Institutes of Health, Bethesda, MD, USA). Target protein levels were normalized to GAPDH.

### 2.6. Immunofluorescence Stainings

For immunofluorescence stainings, cells were grown on cover slips and were fixed in 4% paraformaldehyde in PBS for 15 min. Permeabilization was performed with 0.1% Triton-X-100 in PBS for 10 min. After blocking with 3% BSA in PBS for 20 min, the cells were incubated with the primary antibody anti-CVB3 VP1 (Mediagnost, Reutlingen, Germany) diluted 1:5000 for 1 h. Following incubation with an Alexa-594 (red) labeled goat-anti-mouse antibody F(ab)2 fragment (Life technologies, Carlsbad, CA, USA), diluted 1:4000 for 30 min in the dark, nuclei were counterstained with DAPI (Sigma Aldrich, St. Louis, MO, USA). All steps were performed at room temperature. Images were taken with a Zeiss Axio Imager M2 microscope (Carl Zeiss AG, Oberkochen, Germany).

### 2.7. Electron Microscopy

For electron microscopy, iCell^®^ Cardiomyocytes were infected with MOI 10 of CVB3 for 0 and 8 h, washed three times with medium, fixed for 4 h in 2.5% glutaraldehyde (Paesel & Lorei, Hanau, Germany) in 0.1 M phosphate buffer (pH 7.4), scraped from the cell culture dish, and pelleted. The pellets were washed with phosphate buffer, postfixed in 1% OsO4 in phosphate buffer for 1 h, dehydrated in an ascending series of ethanol and propylene oxide, bloc stained in uranyl acetate for 4 h, and embedded in Araldite (Serva, Germany). Using an ultramicrotome (Ultracut, Leica, Bensheim, Germany), semithin (1 μm) and ultrathin (50 nm) sections were cut. Ultrathin sections were stained with lead citrate, mounted on copper grids, and finally analyzed with a Zeiss EM 10 electron microscope (Oberkochen, Germany). Electron microscopy from non-infected and CVB3-infected mouse hearts was obtained from previous experimental studies [21].

### 2.8. LIVE/DEAD Staining 

For analysis of cell membrane integrity, iCell^®^ Cardiomyocytes were grown on cover slips, infected with MOI 10 for 8 h. Cells were fixed in 4% paraformaldehyde in PBS for 15 min, and subsequently stained with LIVE/DEAD Fixable Red Dead Cell Stain (Thermo Fisher Scientific, Waltham, MA, USA) according to the manufacturer’s protocol.

### 2.9. Transfection with pIRES-EGFP-2A and pIRES-EGFP-3C

iCell^®^ Cardiomyocytes were grown in 6-well plates and transfected with the plasmids pIRES-EGFP-2A and pIRES-EGFP-3C, using the X-tremeGENE HP DNA Transfection Reagent (Hoffmann-La Roche, Basel, Switzerland) according to the manufacturer’s instructions. Transfection was verified by detection of GFP expression using Western blot.

### 2.10. Statistical Analyses 

Statistical analysis was performed with SPSS 24.0 software (IBM Corp., Armonk, NY, USA). When statistical analysis was performed, data are presented as mean ± SD.

## 3. Results

### 3.1. Viral Load and CVB3 Genome Localization in Different CVB3-Infected Cell Lines

**Figure 1:**
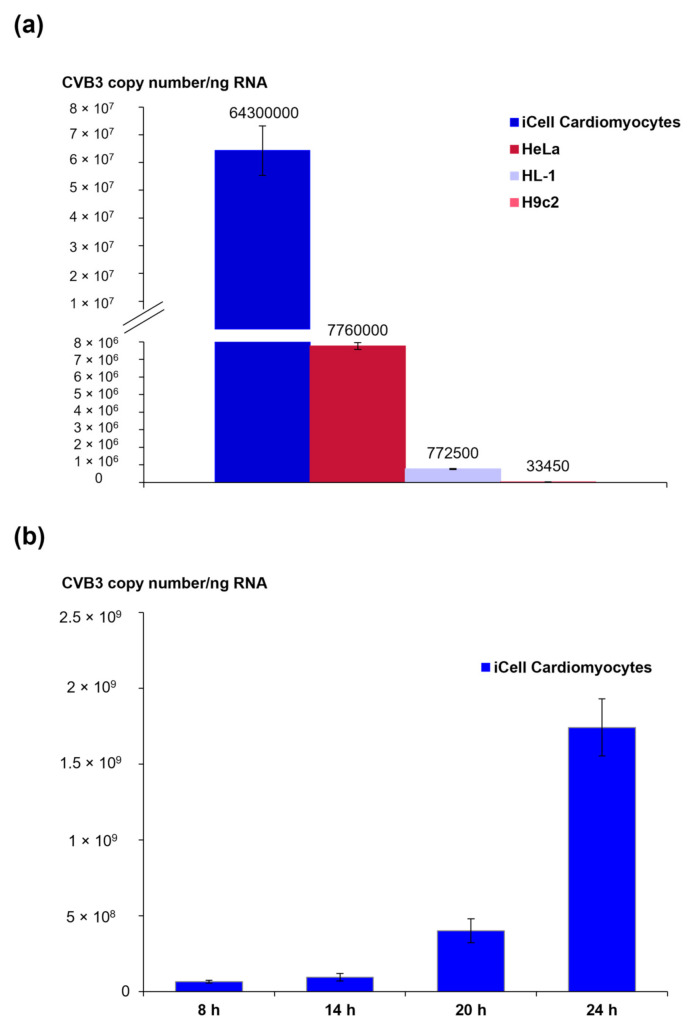
(**a**) Comparison of CVB3 load in different cell lines. CVB3 RNA copy numbers per ng RNA were obtained by qRT-PCR in CVB3-infected HeLa cells, HL-1 cells, H9c2 cells and iCell^®^ Cardiomyocytes, eight hours pi. (**b**) Quantification of CVB3 RNA copy number per ng RNA by qRT-PCR in CVB3-infected iCell^®^ Cardiomyocytes in the time course of infection.

**Figure 2:**
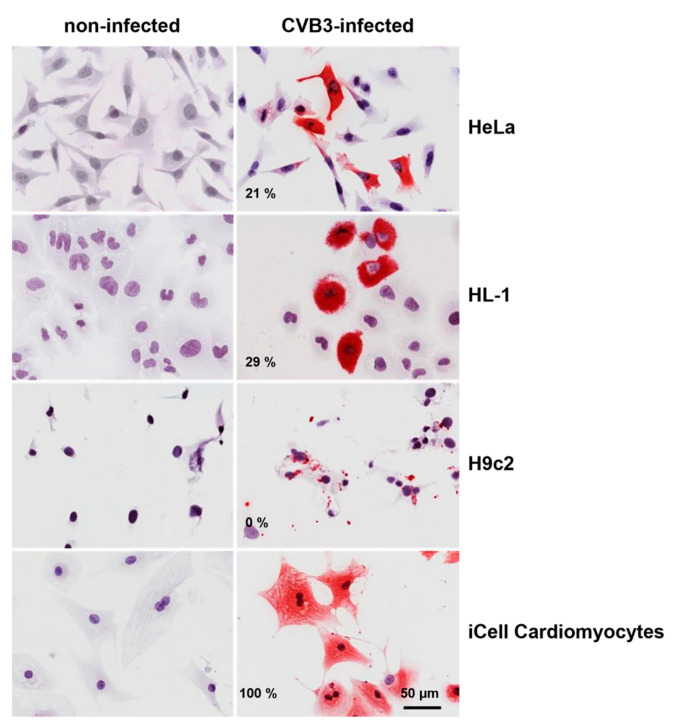
RNA/RNA in situ hybridization for CVB3 RNA localization in different infected cell lines. Cells were infected with a multiplicity of infection (MOI) of 10 and analyzed after eight hours. The percentage of infected cells is indicated in the left lower corner, respectively.

To assess the infection efficacy, the viral load in CVB3-infected iCell^®^ Cardiomyocytes was compared with three cell lines that are commonly used to study the mechanisms of CVB3 infection. RNA was isolated eight hours pi from CVB3-infected HeLa cells, HL-1 cells, H9c2 cells and iCell^®^ Cardiomyocytes, and the viral genome copy number per ng RNA was quantified by qRT-PCR. The highest CVB3 genome copy number was found in infected iCell^®^ Cardiomyocytes and was enhanced compared to all other investigated cell lines (Figure 1a). A time course analysis of the viral load in iCell^®^ Cardiomyocytes (Figure 1b) revealed an exponential increase of viral RNA genomes until 24 h pi, indicating a highly efficient viral replication. A CVB3 genome copy number of approximately 6300 CVB3 per cell was determined for iCell^®^ Cardiomyocytes 24 h pi. CVB3 genome localization in the different CVB3-infected cell lines was evaluated by RNA/RNA in situ hybridization eight hours pi (Figure 2). In all investigated cell lines, CVB3 genomes could be detected, but the percentage of infected cells differed clearly. While iCell^®^ Cardiomyocytes were infected to almost 100%, HeLa cells, HL-1 cells and H9c2 cells exhibited lower infection rates. Interestingly, different staining patterns were observed. In CVB3-infected HeLa cells, HL-1 cells and iCell^®^ Cardiomyocytes, the whole cytoplasm showed intense staining for the presence of CVB3 RNA, whereas in H9c2 only very few small areas of viral replication were found. 

### 3.2. CVB3 Capsid Protein VP1 Expression and Localization in Different CVB3-Infected Cell Lines

**Figure 3:**
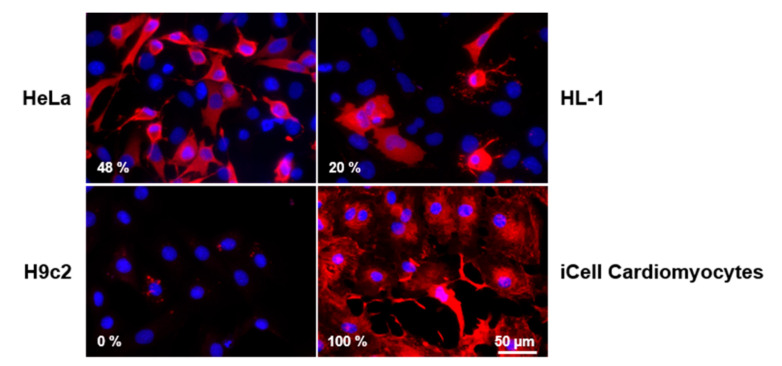
Immunofluorescence analysis of VP1 protein localization in different CVB3-infected cell lines. Cells were infected with MOI 10 and analyzed after eight hours. The percentage of VP1 positively stained cells is indicated in the left lower corner, respectively.

CVB3 infection is frequently proven by analysis of CVB3 capsid protein VP1 expression using either Western blot or immunofluorescence staining [19,22]. Viral protein synthesis, including the synthesis of the viral capsid proteins, is essential for the generation of new virions within the host cell. Therefore, detection of CVB3 capsid protein VP1 expression can be regarded as a reliable indicator for viral protein synthesis. CVB3 VP1 protein localization was also studied by immunofluorescence staining (Figure 3). VP1 protein was detected in nearly 100% of CVB3-infected iCell^®^ Cardiomyocytes, whereas the rate of VP1-positive cells was clearly lower for CVB3-infected HeLa and HL-1 cells (approximately 50% and 20% VP1-positive cells, respectively). HeLa cells, HL-1 cells and iCell^®^ Cardiomyocytes revealed VP1 staining of the whole cytoplasm, whereas H9c2 cells showed only a few positively stained small spots, without evidence of viral protein synthesis within the cytoplasm. While viral protein synthesis could be confirmed for HeLa cells, HL-1 cells and iCell^®^ Cardiomyocytes, the results in H9c2 cells indicate that CVB3 protein synthesis does not efficiently take place in H9c2 cells.

### 3.3. Morphological Changes in the Course of CVB3 Infection in Different Cell Lines

**Figure 4:**
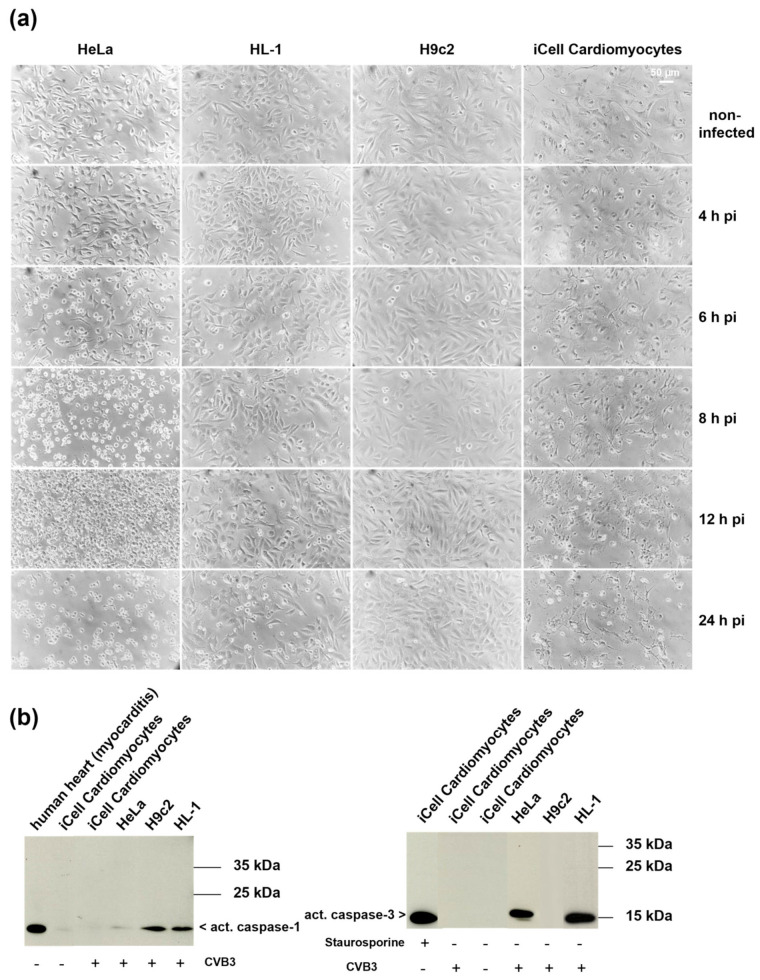
(**a**) Course of CVB3 infection in different cell lines. Brightfield images of CVB3-infected HeLa cells, HL-1 cells, H9c2 cells and iCell^®^ Cardiomyocytes. Cells were infected with MOI 10 and observed for 24 h. (**b**) Western blot analysis of caspase-1 (left panel, positive control: lysate of human heart tissue of a patient with myocarditis) and caspase-3 cleavage (right panel, positive control: iCell Cardiomyocytes treated with staurosporine) in different CVB3-infected cell lines eight hours pi (MOI 10).

The morphological and structural changes caused by viruses are referred to as cytopathic effect (CPE). The extent and characteristics of CPE depend on the respective virus, cell type and species [23]. To detect possible differences regarding the CPE HeLa cells, HL-1 cells, H9c2 cells and iCell^®^ Cardiomyocytes were infected with a multiplicity of infection (MOI) of 10 and observed over a time course (4–24 h pi) (Figure 4a). The CVB3-infected cell lines displayed clear differences regarding the CPE. While no morphological changes were observed in CVB3-infected H9c2 cells at any time of infection, a typical virus-induced CPE with varying onset was noted in the other cell lines. First signs of cellular deformation with protrusions and detachment were found in HeLa cells and iCell^®^ Cardiomyocytes as early as six hours pi, and in HL-1 cells eight hours pi. Rounding and detachment of cells was observed for HeLa cells eight hours pi, whereas HL-1 cells and iCell^®^ Cardiomyocytes did not appear rounded at this time point. Instead, iCell^®^ Cardiomyocytes exhibited signs of vacuolization and cellular lengthening and narrowing. Twelve hours pi, almost all HeLa cells were rounded and detached from the culture vessel, whereas dissolution could be observed for iCell^®^ Cardiomyocytes. At 24 h pi, the cell number was clearly reduced for HeLa cells and iCell^®^ Cardiomyocytes cells, whereas the cell number of the HL-1 cells was slightly reduced, and that of H9c2 was not changed at all (Figure 4a).

### 3.4. Analysis of Cell Death Markers in Different CVB3-Infected Cell Lines 

We found that the CPE observed in the various cell lines clearly differed regarding onset and extent. As contradictory results have also been published concerning the type of cell death caused by CVB3, we wanted to investigate the mode of cell death in CVB3-infected HeLa cells, HL-1 cells, H9c2 cells and iCell^®^ Cardiomyocytes. Among the different modes of cell death, apoptosis and pyroptosis are discussed in the context of CVB3-induced myocarditis [8,9,10,16,24]. To evaluate whether caspase-1 or caspase-3 are activated upon CVB3 infection, we performed Western blot analyses. Activation of caspase-1, which is part of the inflammasome complex, is a characteristic step of pyroptosis [25]. Caspase-3 is involved in the execution phase of apoptosis and is responsible for the cleavage of several cellular targets [26]. As shown in Figure 4b (left panel), caspase-1 was found to be active in CVB3-infected HL-1 and H9c2 cells eight hours pi, but was neither detected in CVB3-infected HeLa cells nor iCell^®^ Cardiomyocytes. In contrast, expression of active caspase-3 could only be verified for CVB3-infected HeLa cells and HL-1 cells (Figure 4b), right panel). These results already indicate activation of different pathways in the individual cell lines upon CVB3 infection. iCell^®^ Cardiomyocytes and HeLa cells clearly differ regarding activation of caspase-3 upon CVB3-activation. In addition, apoptosis has been described as a mode of cell death for CVB3-infected HeLa cells in several publications [27,28].

### 3.5. Comparison of CVB3-Infected iCell^®^ Cardiomyocytes and Murine Heart Tissue of CVB3-Infected Mice by Electron Microscopy

**Figure 5:**
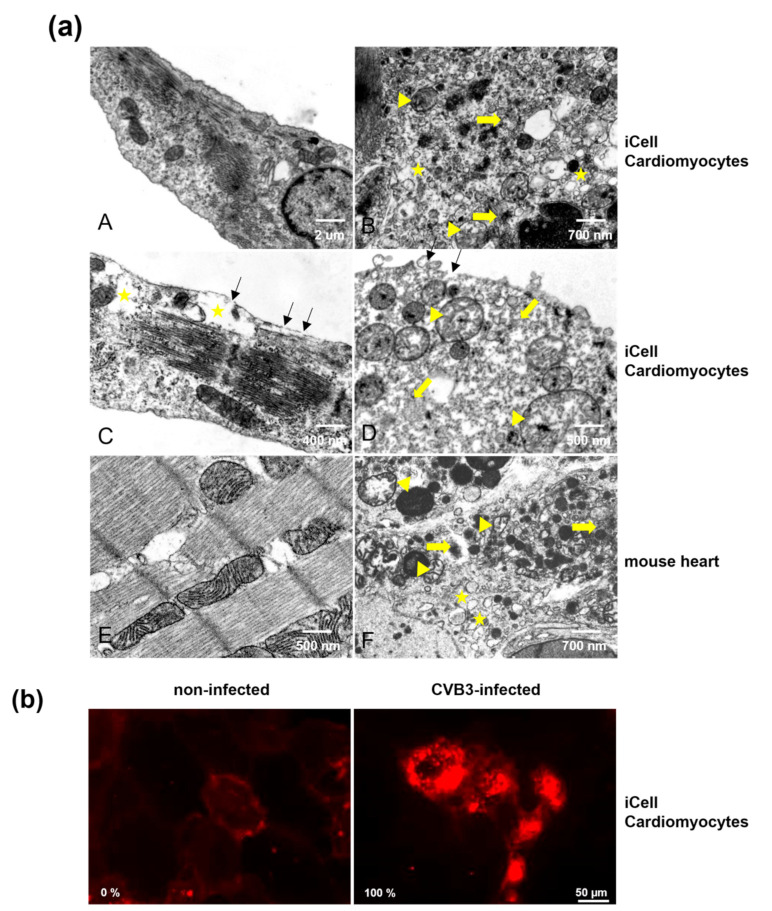
(**a**) Electron microscopy images of iCell^®^ Cardiomyocytes (A, non-infected, B–D infected) and murine heart tissue (E, non-infected; F, infected). iCell^®^ Cardiomyocytes were analyzed eight hours post CVB3 infection (MOI 10) and compared with murine heart tissue of CVB3-infected mice obtained during acute infection (eight days pi). Yellow arrows indicate destruction of myofibrils; yellow triangles indicate structural changes in mitochondria; yellow asterisks indicate the evolvement of vesicular structures; black arrows (C, D) indicate the destruction of the cellular membrane, which was observed in > 90% of 100 analyzed cells. D illustrates end-stage infection with final cellular lysis. (**b**) LIVE/DEAD staining of iCell^®^ Cardiomyocytes with an amine-reactive dye for analysis of cell membrane integrity. Cells were infected with MOI 10 and analyzed after eight hours. The percentage of positively stained cells is indicated in the left lower corner, respectively.

The mouse model of CVB3-induced myocarditis is well established as it best reflects the pathogenesis of myocarditis in humans [29]. To evaluate whether the morphological changes observed in CVB3-infected iCell^®^ Cardiomyocytes mirror those of murine CVB3-infected cardiomyocytes in vivo, we analyzed CVB3-infected iCell^®^ Cardiomyocytes by electron microscopy and compared them with murine heart tissue from acutely CVB3-infected mice [21]. As already described in vivo [21], a loss of host cell integrity with the destruction of cellular membranes (black arrows), myofibrils (yellow arrows), structural changes in mitochondria (yellow arrowheads) and evolvement of vesicular structures (asterisks), reflecting viral replication, were noted for CVB3-infected iCell^®^ Cardiomyocytes (Figure 5a). These results indicate that in iCell^®^ Cardiomyocytes, the virus-induced cytopathic effects reflect the site of viral replication, and thus well represent the findings in the natural host in the heart (the cardiomyocytes).

### 3.6. Analysis of Cell Membrane Integrity of CVB3-Infected iCell^®^ Cardiomyocytes

Neither activation of caspase-1 nor activation of caspase-3 was detected in CVB3-infected iCell^®^ Cardiomyocytes, indicating that CVB3-infected iCell^®^ Cardiomyocytes do not undergo apoptosis or pyroptosis. To gain additional information in the mode of cell death that occurs in CVB3-infected iCell^®^ Cardiomyocytes, we analyzed cell membrane integrity. The loss of the cell membrane integrity, which is characteristic for necrosis, was analyzed by LIVE/DEAD staining with an amine-reactive dye. While cells with an intact cell membrane are stained only weakly on the surface, cells with disturbed membrane integrity present strong staining of the entire cell. The strong intracellular staining of CVB3-infected iCell^®^ Cardiomyocytes and the high percentage (100%) of positively stained cells clearly demonstrates that cell membrane integrity of iCell^®^ Cardiomyocytes is lost upon CVB3 infection (Figure 5b).

### 3.7. The Role of RIPK1 and RIPK3 in CVB3-Infected iCell^®^ Cardiomyocytes

**Figure 6:**
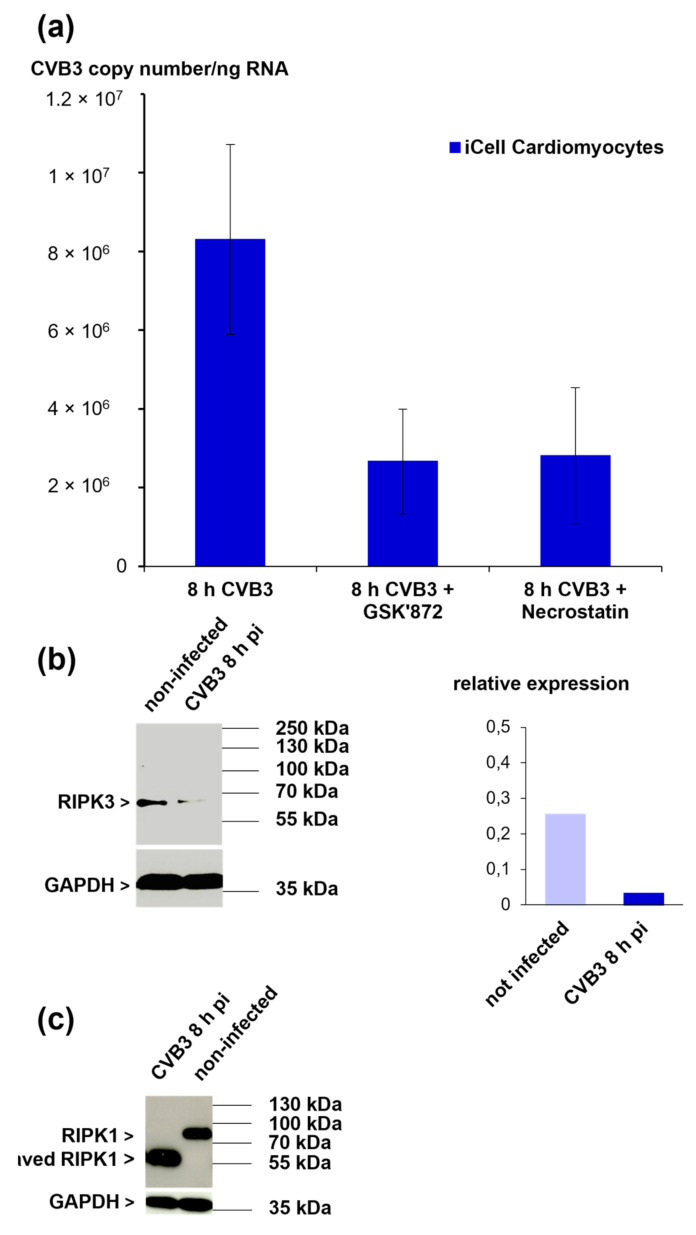
(**a**) Quantification of viral load in iCell^®^ Cardiomyocytes after inhibition of RIPK3 kinase activity (GSK’872) and inhibition of RIPK1 kinase activity (necrostatin). (**b**) Western blot analysis of RIPK3 expression in CVB3-infected iCell^®^ Cardiomyocytes eight hours pi (MOI 10) (left panel). Densitometric analysis is shown in the right panel. Data were normalized to GAPDH. (**c**) Western blot analysis of RPK1 expression in CVB3-infected iCell^®^ Cardiomyocytes eight hours pi (MOI 10).

The observation of a disturbed cell membrane integrity and intracellular morphology in iCell^®^ Cardiomyocytes’ indicates that CVB3-infected iCell^®^ Cardiomyocytes undergo necrosis. However, not only necrosis but also necroptosis (programmed necrosis) is characterized by the destruction of the cell membrane. The role of necroptosis was already discussed in the context of viral myocarditis in the mouse model [11]. To figure out whether necroptosis occurs in CVB3-infected iCell^®^ Cardiomyocytes, the necroptosis key players’ activity (RIPK1 and RIPK3) was inhibited with specific inhibitors and the effect on viral replication was studied. Pre-treatment of iCell^®^ Cardiomyocytes for one hour prior to CVB3 infection with either GSK‘872 (RIPK3-inhibitor) or necrostatin (RIPK1-inhibitor) led to a reduction of viral replication, indicating that the activity of both kinases is required for efficient replication of CVB3 in iCell^®^ Cardiomyocytes (Figure 6a). Furthermore, the expression of RIPK3 and RIPK1 was analyzed by Western blot and revealed a decrease of RIPK3 expression eight hours pi (Figure 6b). Interestingly, for RIPK1, a band with a lower molecular weight (approximately 55 kDa) was observed in CVB3-infected iCell^®^ Cardiomyocytes, suggesting that RIPK1 is cleaved during CVB3 infection of iCell^®^ Cardiomyocytes (Figure 6c).

### 3.8. The Viral Proteases 2Apro and 3Cpro Are Not Responsible for the Cleavage of RIPK1 in iCell^®^ Cardiomyocytes

**Figure 7:**
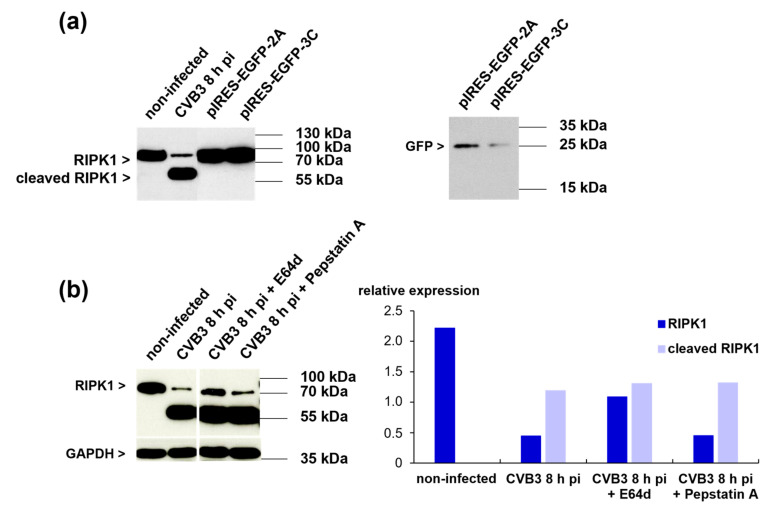
(**a**) Western blot analysis of RIPK1 expression in iCell^®^ Cardiomyocytes transfected with either pIRES-EGFP-2A or pIRES-EGFP-3C (left panel). GFP expression is shown in the right panel. (**b**) Western blot analysis of RIPK1 expression in CVB3-infected iCell^®^ Cardiomyocytes eight hours pi (MOI 10) after pre-incubation with the protease inhibitors E64d or pepstatin A. iCell^®^ Cardiomyocytes were pre-incubated for one hour with the inhibitors before CVB3 infection. Densitometric analysis is shown in the right panel. Data were normalized to GAPDH.

The CVB3 proteases 2Apro and 3Cpro are involved in the maturation of the viral polyprotein, but degrade cellular targets as well [30,31]. Blom et al. established the NetPico RNA 1.0 Server for the prediction of the picornaviral proteases’ cleavage sites [32]. By means of this tool, three possible 3Cpro-cleavage sites were predicted for RIPK1. However, no 2Apro-cleavage sites were predicted. To analyze whether RIPK1 is possibly cleaved by one of the two proteases, we transfected iCell^®^ Cardiomyocytes with plasmids expressing both 2Apro and 3Cpro (pIRES-EGFP-2A and pIRES-EGFP-3C). But neither expression of 2Apro nor of 3Cpro led to cleavage of RIPK1 in iCell^®^ Cardiomyocytes (Figure 7a, left panel). Transfection efficiency was confirmed by analysis of GFP expression (Figure 7a, right panel).

### 3.9. RIPK1 Cleavage Cannot Be Prevented by Protease Inhibitors but Is Inhibited by Treatment with DMSO

**Figure 8:**
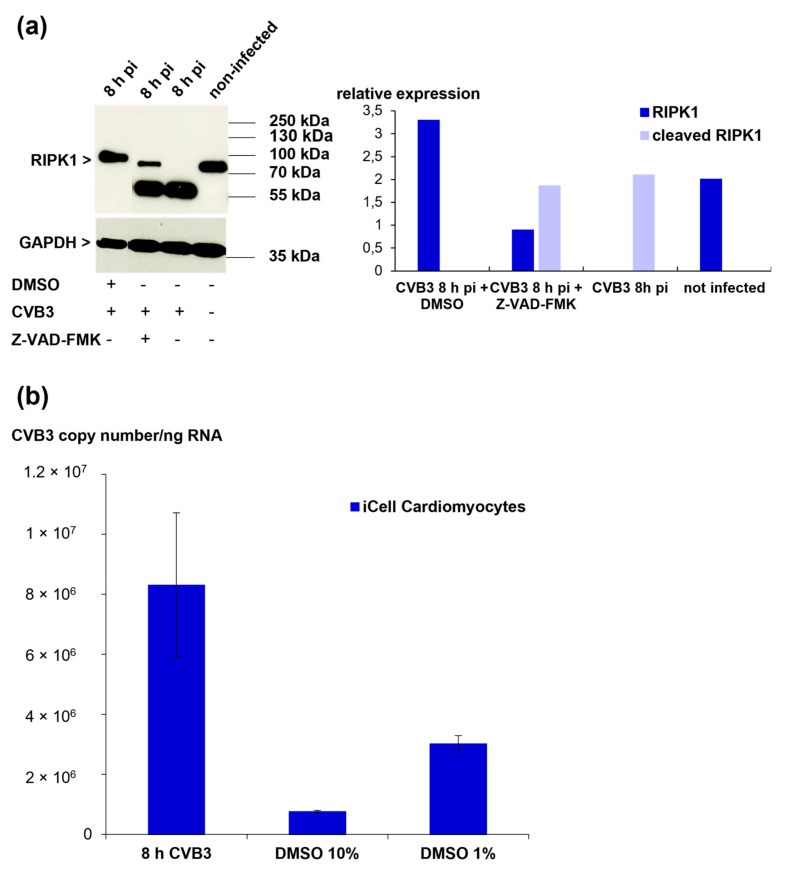
(**a**) Western blot analysis of RIPK1 expression in CVB3-infected iCell^®^ Cardiomyocytes eight hours pi (MOI 10) after pre-treatment with DMSO or Z-VAD-FMK (pan-caspase inhibitor) (left panel). Densitometric analysis is shown in the right panel. Data were normalized to GAPDH. (**b**) Quantification of viral load after DMSO treatment. iCell^®^ Cardiomyocytes were pre-incubated with DMSO at different concentrations for one hour before CVB3 infection.

As we found that RIPK1 cleavage is not executed by the viral proteases 2Apro and 3Cpro, we next asked whether cellular proteases are involved in this process. To address this question, iCell^®^ Cardiomyocytes were pre-incubated for one hour with different protease-inhibitors (E64d, a cysteine protease inhibitor; pepstatin A, an aspartic protease inhibitor; and Z-VAD-FMK, a pan-caspase inhibitor) prior to CVB3 infection. None of these inhibitors was able to prevent the RIPK1 cleavage during CVB3 infection of iCell^®^ Cardiomyocytes (Figure 7b and Figure 8a). Interestingly, we observed that RIPK1 cleavage was prevented in a sample of iCell^®^ Cardiomyocytes that was only treated with 10% DMSO prior to CVB3 infection. A subsequent analysis of DMSO pre-treatment on the viral load revealed a decrease of the CVB3 copy numbers in iCell^®^ cardiomyocytes, suggesting that DMSO treatment has a negative impact on viral replication (Figure 8b). Different concentrations of DMSO were tested to investigate whether the observed effect is only the result of the cytotoxic characteristics of DMSO. Since a final DMSO concentration of 1% still led to a reduction of viral replication, this possibility could be reduced. So far, the mechanisms by which DMSO interferes with virus replication are unclear.

## 4. Discussion

Cardiomyocytes represent the main target cells of CVB3 in the human heart [21]. Thus, the molecular mechanisms occurring in CVB3 myocarditis should therefore preferably be studied in primary human cardiomyocytes. As these cells are very difficult to obtain and can be kept in culture only for a very limited time, researchers utilize different immortalized cell lines as substitutes in in vitro model systems. The fact that cardiomyocytes can now be differentiated from hiPSCs opens up new possibilities in heart research. In this study, we compared a new in vitro model system for CVB3-induced myocarditis, iCell^®^ Cardiomyocytes, with three other cell lines that are commonly used to study CVB3-induced myocarditis. We were able to document great differences between the cell lines regarding infection efficiency, viral load, CPE and mode of cell death.

Our results demonstrate that iCell^®^ Cardiomyocytes are most efficiently infected with CVB3 in comparison to the other cell lines investigated here. For iCell^®^ Cardiomyocytes, an infection rate of almost 100% was noted. This finding was proven by the presence of CVB3 RNA genomes by RNA/RNA in situ hybridization, as well as localization of viral capsid protein VP1 using immunofluorescence staining. The distribution of viral RNA within this cell type perfectly reflects the patterns in cardiomyocytes of CVB3-infected mice [20]. Furthermore, the highest CVB3 RNA genome copy number was detected by qRT-PCR in this cell line. The other cell lines demonstrated infection rates of only 30–50% up to eight hours pi. Interestingly, H9c2 cells seemed not to be properly infected at all. The visualization of CVB3 RNA and VP1 demonstrated for HeLa cells, as well as HL-1 cells, mixed populations of infected and non-infected cells. The possibility that “non-infected” cells are “persistently” infected cells, as described by Pinkert et al., can be excluded by the absence of viral RNA in these cells [33]. Interestingly, Feuer et al. were able to show that quiescent HeLa cells were protected from viral-mediated CPE and suggested that CVB3 replication depends on the cell’s cell cycle status at the time point of infection [34,35]. This observation could also play a role in CVB3 infection of HL-1 cells. Thus, HeLa cells and HL-1 cells would have to be cell cycle-synchronized prior to CVB3 infection to ensure higher infection rates; otherwise, the percentage of infected cells is rather low, which falsifies the results if cell lysates are prepared, e.g., for Western blots. In conclusion, both cell lines’ suitability as an in vitro model system to study CVB3-induced myocarditis is limited. Quantification of the viral load in CVB3-infected H9c2 cells revealed very low CVB3 RNA genome copy numbers compared to the other cell lines. Furthermore, the detection of CVB3 RNA by in situ hybridization revealed a completely different staining pattern compared to the other cell lines, with only a few positive “spots”. This staining pattern was confirmed by the localization of the viral capsid protein VP1, and it is possible that the virus enters the cells but does not replicate in measurable amounts. To summarize, the use of H9c2 cells as an in vitro model system for CVB3-induced myocarditis is even more discouraged than that of HeLa cells and HL-1 cells.

In our experiments, we found that the CPE in the various cell lines clearly differed regarding onset and extent. Regarding the analysis of cell death markers by Western blot, we found activation of the pyroptosis marker caspase-1 in HL-1 cells, whereas active caspase-3, an apoptosis marker, was detected in HL-1 cells and HeLa cells post infection. The activation of caspase-3 in CVB3-infected HeLa cells was already observed by Carthy et al. and Yuan et al. [3,36]. In CVB3-infected HL-1 cells, activation of caspase-1 and of caspase-3 was found. These contradictory results may be explained by the mixed population of infected and non-infected cells; possibly, different signaling cascades are activated in infected and non-infected cells. Non-infected cells may furthermore be affected by molecules secreted by infected cells and suffer from the infection as “bystanders”. As each cell line revealed its own characteristics for cell death marker, our results indicate that the cell lines undergo different forms of cell death and activation of various associated molecular pathways upon CVB3 infection and are not comparable in this regard. Time course experiments regarding the expression of the different cell death markers may be conducted as future experiments to clarify the differences in onset of the different pathways for each cell line.

The analysis of the mode of cell death of CVB3-infected iCell^®^ Cardiomyocytes did not provide evidence that these cells undergo apoptosis or pyroptosis. In contrast, our observations of the changes in the cellular morphology by electron microscopy, as well the loss of cell membrane integrity, indicate that these cells undergo either necrosis or necroptosis, which are both characterized by destruction of the cell membrane [37]. Recent research already discussed the role of necroptosis in the context of viral myocarditis in the mouse model. Zhou et al. found high expression of RIPK1/RIPK3 in the mouse model of acute viral myocarditis and treatment with the RIPK1 inhibitor necrostatin led to reduced myocardial damage [11]. Our experiments revealed that inhibition of RIPK1, as well as inhibition of RIPK3, led to a reduction of viral replication of approximately 50%, indicating that activity of both kinases is required for viral replication. For intestinal epithelial cells, it was already shown that RIPK3 functions as a positive regulator of CVB3 replication, playing a role in regulation of autophagy, a process that is utilized by CVB3 for replication [22,38]. Although RIPK3 may be involved in the facilitation of CVB3 replication, it is nevertheless a key player of the necroptosis pathway, which may be detrimental to CVB3 replication, especially if the cells die too early before replication is successfully completed. Analysis of RIPK1 and RIPK3 protein expression revealed a reduction of RIPK3 expression eight hours pi, whereas RIPK1 was cleaved, indicating that formation of the necrosome is impossible. As expression of 2Apro and 3Cpro in iCell^®^ Cardiomyocytes did not result in cleavage of RIPK1, it seems that RIPK1 cleavage in CVB3-infected iCell^®^ Cardiomyocytes is not mediated by the viral proteases 2Apro and 3Cpro. Cleavage of RIPK1 by different caspases has already been described for the prevention of necroptosis [39,40,41]. Pre-incubation of iCell^®^ Cardiomyocytes with the pan-caspase inhibitor Z-VAD-FMK did not prevent RIPK1 cleavage. Additionally, application of E64d, a cysteine protease inhibitor, as well as Pepstatin A, an aspartate protease inhibitor, did not prevent RIPK1 cleavage. Interestingly, pre-incubation with DMSO prior to CVB3 infection prevented RIPK1 cleavage and led to a reduction of viral replication. This effect was observed with a DMSO concentration of 10% but also with a lower concentration of only 1% DMSO, decreasing the possibility that the observed effect is only due to the cytotoxic characteristics of DMSO. Aguilar et al. demonstrated that even treatment of cells with 5% DMSO for eight hours did not result in a significant reduction of cell viability [42]. Although it cannot be excluded completely that the observed effect is, at least in part, due to the cytotoxic characteristics of DMSO, similar results have been obtained for other viruses. Importantly, for herpes simplex virus-1 (HSV-1), it was shown that DMSO blocks the productive infection with this virus. Here, distinct temporal target mechanisms were found, including reduction of virion infectivity, inhibition of viral DNA replication and reduction of transcription levels of HSV-1 genes [42]. Furthermore, it was shown that DMSO inhibits human herpes virus-6 (HHV-6) infection when added to HHV-6-infected HSB2 cultures [43]. The effect of various concentrations of DMSO on influenza A virus (PR-8) infectivity (amongst other viruses) has also been studied, but inactivation of infectivity was only noted at DMSO concentrations higher than 50% [44]. The exact mechanisms by which DMSO interferes with viral replication in iCell^®^ Cardiomyocytes and which role cleavage of RIPK1 plays in this context remain to be elucidated.

In summary, our results indicate that many cell lines typically used in vitro to study CVB3-induced myocarditis considerably differ in their permissiveness to CVB3 infection, patterns of infection, viral load, and mode of cell death, raising doubts about their suitability to investigate CVB3 pathogenesis in the myocardium. For future experiments, it should be kept in mind that results and conclusions that are drawn for one cell line may not be applicable for other cell lines and importantly, may not reflect the in vivo situation in enterovirus-infected hearts of humans and mice. The same is true for results and conclusions of in vitro investigations published years ago in this context. In contrast, our study suggests that iCell^®^ Cardiomyocytes represent an appropriate model system to investigate CVB3-induced myocarditis. iCell^®^ Cardiomyocytes seem to be homogeneously infected with CVB3, reveal a high infection rate, and undergo morphological changes that are observed in cardiomyocytes in the well-established mouse model of CVB3-induced myocarditis. Thus, these cells best represent the natural host of CVB3, the cardiomyocytes, and are most suitable for the study of molecular mechanisms of enteroviral myocarditis. Recently, iCell^®^ Cardiomyocytes were successfully used for investigation of a novel antiviral drug candidate. Yun et al. were able to generate a CVB3 RNA helicase 2C inhibitor, which significantly inhibited the replication of CVB3 and reduced heart damage and inflammation [45]. However, despite such promising applications of iCell^®^ Cardiomyocytes, it must be taken in account that the pathogenesis of CVB3-induced myocarditis is very complex and in vitro models will always have limitations, allowing only the study of certain aspects of this disease.

Limitations of this study:

The present study has several limitations. For analysis of RIPK1 cleavage by the viral proteases 2Apro and 3Cpro, iCell^®^ Cardiomyocytes were transfected with plasmids expressing both 2Apro and 3Cpro (pIRES-EGFP-2A and pIRES-EGFP-3C). Transfection efficiency was only confirmed by analysis of GFP protein expression, since no anti-2Apro- and anti-3Cpro-antibodies are currently purchasable. Additionally, protease activity of 2Apro and 3Cpro has not been tested after transfection. Consequently, it cannot be excluded that 2Apro and 3Cpro were expressed but not active. However, it is well known that the CVB3 proteases 2A and 3C are key players of the viral life-cycle [46]. In human iPS cardiomyocytes, suppression of the enteroviral 2C helicase was found to inhibit CVB3 replication by using an inhibitor which was based on chemically modified structure of the 3C protease inhibitor [45]. Furthermore, while the RIPK inhibitors GSK‘872 and necrostatin have already been characterized extensively by other groups [47,48,49], experiments to test whether these inhibitors are working as intended in the iCell^®^ Cardiomyocytes could have been performed additionally (such as induction of necroptosis by an external stimulus, with and without application of GSK‘872 and necrostatin).

## Data Availability

The datasets used and/or analyzed during the current study are available from the corresponding author upon reasonable request.

## References

[1] Caforio A.L., Pankuweit S., Arbustini E., Basso C., Gimeno-Blanes J., Felix S.B., Fu M., Helio T., Heymans S., Jahns R. (2013). Current state of knowledge on aetiology, diagnosis, management, and therapy of myocarditis: A position statement of the European Society of Cardiology Working Group on Myocardial and Pericardial Diseases. Eur. Heart J..

[2] Pollack A., Kontorovich A.R., Fuster V., Dec G.W. (2015). Viral myocarditis--diagnosis, treatment options, and current controversies. Nature reviews. Cardiology.

[3] Carthy C.M., Granville D.J., Watson K.A., Anderson D.R., Wilson J.E., Yang D., Hunt D.W.C., McManus B.M. (1998). Caspase Activation and Specific Cleavage of Substrates after Coxsackievirus B3-Induced Cytopathic Effect in HeLa Cells. J. Virol..

[4] Claycomb W.C., Lanson N.A., Stallworth B.S., Egeland D.B., Delcarpio J.B., Bahinski A., Izzo N. (1998). HL-1 cells: A cardiac muscle cell line that contracts and retains phenotypic characteristics of the adult cardiomyocyte. Proc. Natl. Acad. Sci. USA.

[5] Li M., Wang X., Yu Y., Yu Y., Xie Y., Zou Y., Ge J., Peng T., Chen R. (2014). Coxsackievirus B3-induced calpain activation facilitates the progeny virus replication via a likely mechanism related with both autophagy enhancement and apoptosis inhibition in the early phase of infection: An in vitro study in H9c2 cells. Virus Res..

[6] Kimes B., Brandt B. (1976). Properties of a clonal muscle cell line from rat heart. Exp. Cell Res..

[7] Bozym R.A., Patel K., White C., Cheung K.H., Bergelson J.M., Morosky S.A., Coyne C.B. (2011). Calcium signals and calpain-dependent necrosis are essential for release of coxsackievirus B from polarized intestinal epithelial cells. Mol. Biol. Cell.

[8] Saraste A., Arola A., Vuorinen T., Kyto V., Kallajoki M., Pulkki K., Voipio-Pulkki L.M., Hyypia T. (2003). Cardiomyocyte apoptosis in experimental coxsackievirus B3 myocarditis. Cardiovasc. Pathol..

[9] Nie J., Ta N., Liu L., Shi G., Kang T., Zheng Z. (2019). Activation of CaMKII via ER-stress mediates coxsackievirus B3-induced cardiomyocyte apoptosis. Cell Biol. Int..

[10] Wu R., Wu T., Li P., Wang Q., Shi Y., Zhan Y., Zhang S., Xia T., Wang Z., Lv H. (2020). The protection effects of survivin in the cell model of CVB3-induced viral myocarditis. Hear. Vessel..

[11] Zhou F., Jiang X., Teng L., Yang J., Ding J., He C. (2017). Necroptosis may be a novel mechanism for cardiomyocyte death in acute myocarditis. Mol. Cell. Biochem..

[12] Vercammen D., Beyaert R., Denecker G., Goossens V., Van Loo G., Declercq W., Grooten J., Fiers W., Vandenabeele P. (1998). Inhibition of Caspases Increases the Sensitivity of L929 Cells to Necrosis Mediated by Tumor Necrosis Factor. J. Exp. Med..

[13] Belizário J., Cordeiro L., Enns S. (2015). Necroptotic Cell Death Signaling and Execution Pathway: Lessons from Knockout Mice. Mediat. Inflamm..

[14] Vandenabeele P., Declercq W., Van Herreweghe F., Berghe T.V. (2010). The Role of the Kinases RIP1 and RIP3 in TNF-Induced Necrosis. Sci. Signal..

[15] Wang H., Sun L., Su L., Rizo J., Liu L., Wang L., Wang F.-S., Wang X. (2014). Mixed Lineage Kinase Domain-like Protein MLKL Causes Necrotic Membrane Disruption upon Phosphorylation by RIP3. Mol. Cell.

[16] Toldo S., Kannan H., Bussani R., Anzini M., Sonnino C., Sinagra G., Merlo M., Mezzaroma E., De Giorgio F., Silvestri F. (2014). Formation of the inflammasome in acute myocarditis. Int. J. Cardiol..

[17] Wang Y., Gao B., Xiong S. (2014). Involvement of NLRP3 inflammasome in CVB3-induced viral myocarditis. Am. J. Physiol. Circ. Physiol..

[18] Mummery C.L. (2018). Perspectives on the Use of Human Induced Pluripotent Stem Cell-Derived Cardiomyocytes in Biomedical Research. Stem Cell Rep..

[19] Sharma A., Marceau C., Hamaguchi R., Burridge P.W., Rajarajan K., Churko J.M., Wu H., Sallam K.I., Matsa E., Sturzu A.C. (2014). Human Induced Pluripotent Stem Cell–Derived Cardiomyocytes as an In Vitro Model for Coxsackievirus B3–Induced Myocarditis and Antiviral Drug Screening Platform. Circ. Res..

[20] Klingel K., Hohenadl C., Canu A., Albrecht M., Seemann M., Mall G., Kandolf R. (1992). Ongoing enterovirus-induced myocarditis is associated with persistent heart muscle infection: Quantitative analysis of virus replication, tissue damage, and inflammation. Proc. Natl. Acad. Sci. USA.

[21] Klingel K., Rieger P., Mall G., Selinka H.C., Huber M., Kandolf R. (1998). Visualization of enteroviral replication in myocardial tissue by ultrastructural in situ hybridization: Identification of target cells and cytopathic effects. Lab. Investig..

[22] Harris K.G., Morosky S.A., Drummond C.G., Patel M., Kim C., Stolz D.B., Bergelson J.M., Cherry S., Coyne C.B. (2015). RIP3 Regulates Autophagy and Promotes Coxsackievirus B3 Infection of Intestinal Epithelial Cells. Cell Host Microbe.

[23] Albrecht T., Fons M., Boldogh I., Rabson A.S., Baron S. (1996). Effects on Cells. Medical Microbiology.

[24] Wang Y., Jia L., Shen J., Wang Y., Fu Z., Su S.-A., Cai Z., Wang J.-A., Xiang M. (2018). Cathepsin B aggravates coxsackievirus B3-induced myocarditis through activating the inflammasome and promoting pyroptosis. PLOS Pathog..

[25] Yuan Y.-Y., Xie K.-X., Wang S.-L., Yuan L.-W. (2018). Inflammatory caspase-related pyroptosis: Mechanism, regulation and therapeutic potential for inflammatory bowel disease. Gastroenterol. Rep..

[26] Elmore S. (2007). Apoptosis: A Review of Programmed Cell Death. Toxicol. Pathol..

[27] Li X., Li Z., Zhou W., Xing X., Huang L., Tian L., Chen J., Chen C., Ma X., Yang Z. (2013). Overexpression of 4EBP1, p70S6K, Akt1 or Akt2 differentially promotes Coxsackievirus B3-induced apoptosis in HeLa cells. Cell Death Dis..

[28] Li X., Zhang J., Chen Z., Yang L., Xing X., Ma X., Yang Z. (2013). Both PI3K- and mTOR-signaling pathways take part in CVB3-induced apoptosis of Hela cells. DNA Cell Biol..

[29] Błyszczuk P. (2019). Myocarditis in Humans and in Experimental Animal Models. Front. Cardiovasc. Med..

[30] Krausslich H.G., Wimmer E. (1988). Viral proteinases. Annu. Rev. Biochem..

[31] Lawson M.A., Semler B.L. (1990). Picornavirus protein processing--enzymes, substrates, and genetic regulation. Curr. Top. Microbiol. Immunol..

[32] Blom N.S., Hansen J., Brunak S., Blaas D. (1996). Cleavage site analysis in picornaviral polyproteins: Discovering cellular targets by neural networks. Protein Sci..

[33] Pinkert S., Klingel K., Lindig V., Dorner A., Zeichhardt H., Spiller O.B., Fechner H. (2011). Virus-Host Coevolution in a Persistently Coxsackievirus B3-Infected Cardiomyocyte Cell Line. J. Virol..

[34] Feuer R., Mena I., Pagarigan R., Slifka M.K., Whitton J.L. (2002). Cell Cycle Status Affects Coxsackievirus Replication, Persistence, and Reactivation In Vitro. J. Virol..

[35] Feuer R., Mena I., Pagarigan R.R., Hassett D.E., Whitton J.L. (2004). Coxsackievirus replication and the cell cycle: A potential regulatory mechanism for viral persistence/latency. Med Microbiol. Immunol..

[36] Yuan J.P., Zhao W., Wang H.T., Wu K.Y., Li T., Guo X.K., Tong S.Q. (2003). Coxsackievirus B3-induced apoptosis and Caspase-3. Cell Res..

[37] Zhang Y., Chen X., Gueydan C., Han J. (2017). Plasma membrane changes during programmed cell deaths. Cell Res..

[38] Mohamud Y., Shi J., Tang H., Xiang P., Xue Y.C., Liu H., Ng C.S., Luo H. (2020). Coxsackievirus infection induces a non-canonical autophagy independent of the ULK and PI3K complexes. Sci. Rep..

[39] Dunai Z.A., Imre G., Barna G., Korcsmáros T., Petak I., Bauer P.I., Mihalik R. (2012). Staurosporine Induces Necroptotic Cell Death under Caspase-Compromised Conditions in U937 Cells. PLoS ONE.

[40] Van Raam B.J., Ehrnhöfer D., Hayden M., Salvesen G.S. (2012). Intrinsic cleavage of receptor-interacting protein kinase-1 by caspase-6. Cell Death Differ..

[41] Lin Y., Devin A., Rodriguez Y., Liu Z.-G. (1999). Cleavage of the death domain kinase RIP by Caspase-8 prompts TNF-induced apoptosis. Genes Dev..

[42] Aguilar J., Roy D., Ghazal P., Wagner E. (2002). Dimethyl sulfoxide blocks herpes simplex virus-1 productive infection in vitro acting at different stages with positive cooperativity. Application of micro-array analysis. BMC Infect. Dis..

[43] Viza D., Aranda-Anzaldo A., Ablashi D., Kramarsky B. (1992). HHV-6 inhibition by two polar compounds. Antivir. Res..

[44] Chan J.C., Gadebusch H.H. (1968). Virucidal properties of dimethyl sulfoxide. Appl. Microbiol..

[45] Yun S.H., Shin H.H., Ju E.S., Lee Y.J., Lim B.K., Jeon E.S. (2020). Inhibition of RNA Helicase Activity Prevents Coxsackievirus B3-Induced Myocarditis in Human iPS Cardiomyocytes. Int. J. Mol. Sci..

[46] Peischard S., Ho H.T., Theiss C., Strutz-Seebohm N., Seebohm G. (2019). A Kidnapping Story: How Coxsackievirus B3 and Its Host Cell Interact. Cell. Physiol. Biochem..

[47] Kaiser W.J., Sridharan H., Huang C., Mandal P., Upton J.W., Gough P.J., Sehon C.A., Marquis R.W., Bertin J., Mocarski E.S. (2013). Toll-like receptor 3-mediated necrosis via TRIF, RIP3, and MLKL. J. Biol. Chem..

[48] Degterev A., Huang Z., Boyce M., Li Y., Jagtap P., Mizushima N., Cuny G.D., Mitchison T.J., Moskowitz M.A., Yuan J. (2005). Chemical inhibitor of nonapoptotic cell death with therapeutic potential for ischemic brain injury. Nat. Chem. Biol..

[49] Degterev A., Hitomi J., Germscheid M., Ch’en I.L., Korkina O., Teng X., Abbott D., Cuny G.D., Yuan C., Wagner G. (2008). Identification of RIP1 kinase as a specific cellular target of necrostatins. Nat. Chem. Biol..

